# Evaluation of the impact of low activity imaging in [^11^C]-(+)-PHNO and [^11^C]UCB-J PET-MR scans

**DOI:** 10.1186/s40658-026-00850-y

**Published:** 2026-03-10

**Authors:** Daniela Ribeiro, William Hallett, Oliver Howes, Robert McCutcheon, Matthew M. Nour, David Nutt, David Erritzoe, Claudio Agnorelli, Stephen Husbands

**Affiliations:** 1 Department of Health, University of Bath, Bath, UK; 2 Perceptive, London, UK; 3 King’s College London, London, UK; 4 Medical Research Council London, Institute of Medical Sciences, London, UK; 5 Imperial College London, London, UK; 6 Oxford University, Oxford, UK; 7 University College London, London, UK

**Keywords:** PET-CT, PET-MR, Injected activity reduction, Injected activity optimisation, Sustainability, Radiation protection

## Abstract

**Introduction:**

Positron Emission Tomography (PET) imaging is a close ally of Precision Medicine, and it has been proven to be indispensable in the field of Psychiatry. This imaging modality may also present an important role in understanding Neurodevelopmental disorders and their link to Psychiatric conditions, with new highly selective binders being used currently in research. PET imaging requires the administration of radiopharmaceuticals, where the radioisotope is in incorporated into a highly selective binder. Dosimetry and injected activity optimisation play a crucial role in the field of PET imaging as they allow to determine the radiation dose absorbed by target and non-target tissues, and determine the lowest amount required to deliver images with diagnostic quality and obtain reliable quantitative data, without overexposing patients. The aim of this research is to investigate the feasibility of reducing the injected activity of the [^11^C]-(+)-PHNO and [^11^C]UCB-J radiopharmaceuticals, for patients with neurodevelopmental disorders who undergo brain imaging in the PET-Magnetic Resonance (MR) scanner, without compromising quantitative accuracy of outcome measures.

**Results:**

No statistically significant differences were found when comparing the 1/2 to 1/6 datasets with the full injected activity [^11^C]-(+)-PHNO dataset. Furthermore, the findings obtained from investigating the impact of low injected activity administrations of [^11^C]UCB-J revealed that it is possible to reduce the administered activity by 1/2, when the clinical outcome measure under evaluation is the binding potential relative to non-displaceable volume (BP_ND_). When the outcome measure under investigation is the standard uptake volume ratio (SUV_R_), it is possible to decrease the injected activity to 1/3, for [^11^C]UCB-J.

**Conclusions:**

The simulation and analysis methodologies deployed in this project are suitable for investigating scans with low injected activity for tracers with cortical and striatal uptake, when the outcome measure assessed is the BP_ND_ or the SUV_R_. Whilst the data suggests that imaging with low injected activity is achievable, the efficacy of the investigation is highly dependent on the algorithm used to reconstruct the images, the outcome measure and the radiopharmaceutical used to acquire the PET-MR scans. For the [^11^C]UCB-J radiopharmaceutical, it is possible to decrease the injectable activity to 1/3 of the original administration without compromising the SUV_R_.

**Supplementary Information:**

The online version contains supplementary material available at 10.1186/s40658-026-00850-y.

## **Background**

 Nuclear Medicine, and more specifically Positron Emission Tomography (PET), is an imaging modality which has been used in the field of Psychiatry with the aim of better understanding the aetiology of certain conditions and improving the quality of life of patients suffering from mental health illnesses [1].

In PET imaging, a highly selective binder designed to bind, with high affinity, to molecular targets is labelled with a radioisotope and administered to patients, with the aim to visualise and quantify target expression in vivo. The high sensitivity of these binders allows them to interact with biomolecules that are expressed or dysregulated in certain diseases or conditions and acquire imaging and quantitative PET data with high reliability. This data can, in turn, be used to stratify patients and select the most appropriate therapy, hence advancing precision medicine. The advance of Precision Medicine as an emerging approach which considers gene variability, environmental and lifestyles factors in the treatment and strategic prevention, has further established the role of PET imaging in the Psychiatric field [2, 3]. 

Whilst optimising the administered activity of radiopharmaceuticals is part of the “As Low As Reasonably Achievable” (ALARA) principals in diagnostic imaging, this is additionally important when conducting PET imaging in Psychiatry, as it is likely that younger individuals will be referred for imaging. Due to their developing tissues and greater cell division rate, young patients are more vulnerable to the effects of radiation, in comparison to adults. The risks arising from radiation exposure are cumulative and therefore may have an impact later in life [4].

Neurodevelopmental disorders (NDDs), in particular, are conditions arising from abnormal brain development and are often characterised by cognitive, communicative and/or behavioural impairments which may affect motor skills [5]. Symptoms related to NDDs have been described since the 18th century however, it wasn’t until the mid-20th century that these disorders began to be classified as discrete entities [6]. Currently, the Diagnostic and Statistical Manual of Mental Disorders fifth edition (DSM-5) recognises conditions within the autism spectrum disorder (ASD), attention deficit hyperactivity disorder (ADHD) and intellectual disability (ID) as NDDs [7].

Evidence however suggests that childhood neurodevelopmental disorders appear to share specific genetic alleles not only with each other but also with psychiatric disorders, with emerging data proposing that there may be pathogenic mechanisms which overlap between ID, ASD and ADHD and bipolar disorder (BPD) and schizophrenia [6]. Moreover, people suffering from NDDs present higher rates of psychiatric morbidity [7].

Imaging patients at earlier timepoints may allow a better understanding of the aetiology of NDDs, and longitudinal imaging may offer insights into the pathogenic mechanisms overlapping between NDDs and psychiatric conditions. Optimisation of injected activities should, therefore, be considered and addressed in psychiatric populations due to the young age of the participants and the potential longitudinal examinations they may require follow-up on the disease process and therapeutic efficacy [8].

The aim of this study was to investigate the feasibility of reducing the injected activity of radiopharmaceuticals in patients with neurodevelopmental disorders who undergo brain imaging in the PET-MR scanner, without compromising quantitative accuracy of outcome measures. To address this, PET-MR datasets with the full injected activity were compared to simulated datasets with low injected activity, and the accuracy of two clinical outcomes measures were assessed through statistical tests.

## **Methods**

A retrospective analysis was performed on data acquired from two academic research studies. Study 1, required the administration of (4aR,10bR)-4-propyl-3,4,4a,5,6,10b-hexahydro-2 H-naphtho[1,2-b] [1, 4] oxazin-9-ol ([^11^C]-(+)-PHNO) which is a radiopharmaceutical that binds to D2 and D3 receptors in the brain [9]. [^11^C]-(+)-PHNO can be used as a surrogate for measuring extracellular dopamine changes, with its normal biodistribution in the brain including the substantia nigra, hypothalamus, ventral striatum, globus pallidus and thalamus [10, 11]. Study 2, required the administration of (4R)-1-{[3-(^11^C)Methylpyridin-4-yl]methyl}-4-(3,4,5-trifluorophenyl)pyrrolidin-2-one ([^11^C]UCB-J) which is a radiopharmaceutical that binds to the synaptic vesicle glycoprotein 2 A in the brain [12]. [^11^C]UCB-J can be used as a marker for synaptic loss, with its normal biodistribution in the brain including the thalamus, ventral striatum, caudate, insula, parietal lobe and frontal cortex [13]. In both studies, counts were acquired dynamically over 90 min, and binned into 31 frames (duration: 8 × 15s, 3 × 60s, 5 × 120s, 15 × 300). This process allows for the evolution of the tracer to be preserved, ensuring the shorter initial frames capture the early phase of the radiopharmaceutical and the longer later frames capture the slower biological phase of the tracer distribution, even during the low activity simulations.

Neither of these radiopharmaceuticals is currently being used in clinical practice. This is mainly due to the short half-life of carbon-11, which does not allow them to be transported as they decay too rapidly.

Both studies adhered to the principles outlined in the National Health Service (NHS) Research Governance Framework for Health and Social Care (2nd edition), the Declaration of Helsinki and Good Clinical Practice (GCP). The data used in this project was acquired after the participants’ consent was obtained for the original study. Use of this data was covered in the original consent form, which stated that the data acquired could be used in future related research and written permission was obtained from both Chief Investigators.

### [^11^C]-(+)-PHNO PET-MR datasets

Datasets from 10 adult healthy participants who had received [^11^C]-(+)-PHNO were retrieved for re-reconstruction and analysis. The average age of the participants was 22.4 years with the female to male ratio being 6:4. The mean administered activity was 134.2 ± 20.3 MBq (mean ± SD, *n* = 10).

The ZTE sequence was acquired and used for attenuation correction of the PET images. The T1-weighted images were acquired as Bravo sequences, to assist with image analysis.

### [^11^C]UCB-J PET-MR datasets

Datasets from 5 healthy adult participants who had received [^11^C]UCB-J were retrieved for re-reconstruction and analysis. The average age of the participants was 38.6 years, with only males participating in the study. The mean administered activity was 228.6 ± 58.3 MBq (mean ± SD, *n* = 5).

The ZTE sequence was acquired and used for attenuation correction of the PET images. The T1-weighted images were acquired as fast spoiled gradient-echo (FSPGR) sequences, to assist with image analysis.

### Low data simulations and reconstructions

As the original data was acquired in a list-mode format, low-activity datasets were generated by introducing delays at the start of each frame and re-reconstructing the data contained within the last section of each frame. This approach ensured that the data binned at the start of the frame was consistently rejected and the data binned at the end of the frames was consistently included.

Seven low activity simulations were simulated: 1/2, representing 50%; 1/3, representing 33%; 1/4, representing 25%; 1/5, representing 20%; 1/6, representing 16.66%; 1/10, representing 10%; and 1/15 representing 6.67% of the original administered activity. The simulated data was binned according to the framework indicated in Table 1:

**Table 1:** Frame and duration scheme to simulate low activity datasets

	Start of the acquisition			End of the acquisition
Full activity	8 frames x 15 s	3 frames x 60 s	5 frames x 120 s	15 frames x 300 s
1_2_LowActivity	8 × 7s (8s delay)	3 × 30s (30s delay)	5 × 60s (60s delay)	15 × 150s (150s delay)
1_3_LowActivity	8 × 5s (10s delay)	3 × 20s (40s delay)	5 × 40s (80s delay)	15 × 100s (200s delay)
1_4_LowActivity	8 × 4s (11s delay)	3 × 15s (45s delay)	5 × 30s (90s delay)	15 × 75s (225s delay)
1_5_LowActivity	8 × 3s (12s delay)	3 × 12s (48s delay)	5 × 24s (96s delay)	15 × 60s (240s delay)
1_6_LowActivity	8 × 2s (13s delay)	3 × 10s (50s delay)	5 × 20s (100s delay)	15 × 50s (250s delay)
1_10_LowActivity	8 × 1s (14s delay)	3 × 6s (54s delay)	5 × 12s (108s delay)	15 × 30s (270s delay)
1_15_LowActivity	8 × 1s (14s delay)	3 × 4s (56s delay)	5 × 8s (112s delay)	15 × 20s (280s delay)

Simulating dose reduction by introducing delays reflects realistic clinical conditions in which a reduced administered activity results in a lower number of detected coincidences. This strategy allows for the statistical and temporal characteristics of the scanner to be maintained. Moreover, this method allows use of the standard clinical reconstruction workflow without the need for dedicated list-mode manipulation tools, thereby guaranteeing that the methodology deployed can be reproduced in different scanners.

All PET data was reconstructed with a display field of view (DFOV) of 30 cm and a matrix of 192 × 192. An OSEM algorithm with time-of-flight information (TOF) denominated VPFXS was used with 6 iterations, 16 subsets, a 5 mm filter in the xy-axis and no filter in the z-axis.

### Image analysis

For the [^11^C]-(+)-PHNO datasets, the impact of the low activity simulations was investigated on the binding potential relative to non-displaceable volume (BP_ND_) outcome measure for the striatum, caudate, putamen, globus pallidus, thalamus, substantia nigra, accumbens and cerebellum. A single reference tissue model (SRTM) with the cerebellum as a reference region was used. This was due to the cerebellum representing a region of the brain devoid of receptors that would bind to [^11^C]-(+)-PHNO [14].

For the [^11^C]UCB-J datasets, the impact of the low activity simulations was investigated on the BP_ND_ and Standard Uptake Volume ratio (SUV_R_) outcome measures for the brainstem, substantia nigra, thalamus, striatum, caudate, putamen, hippocampus, insular cortex, temporal lobe, parietal lobe, frontal cortex, cerebellum and accumbens. A SRTM model with the centrum semiovale as reference region was used. This was due to the low binding of [^11^C]UCB-J to white matter and previous research having determined that the centrum semiovale is a suitable pseudo reference region [13].

All images were analysed using a PET quantification software denominated MIAKAT. The pipeline follows a sequence of steps namely brain extraction (using MATLAB and FSL functions), brain segmentation (using SPM12 function), motion correction, region of interest (ROI) definition, ROI tracer kinetic modelling and parametric imaging. The outputs of each step were reviewed and manually accepted by the investigator.

### Data analysis

Descriptive statistics were calculated for each structure, per simulation. Similarly, the coefficient of variation (CV) (which indicates the dispersion of the data acquired for all participants in relation to the mean, per dataset, per brain structure), Bland-Altman plots (which demonstrate the relationship between the full activity-simulation paired variables, per brain structure) and bias (which indicates the difference between the outcome measure obtained from the simulation and the outcome measure from the full activity dataset, per brain structure) were also calculated [15, 16]. These statistics were calculated using the GraphPad Prism version 9 software. Normality and Homogeneity of variance were investigated with the Shapiro Wilko and Levene’s test, respectively. The ANOVA with Bonferroni multicomparisons and the Kruskal-Wallis tests were used to investigate statistically significant differences. These were performed using IBM SPSS Statistics software, version 29.0.1.1.

## Results

### Findings of low activity [^11^C]-(+)-PHNO on BP_ND_

**Fig. 1:**
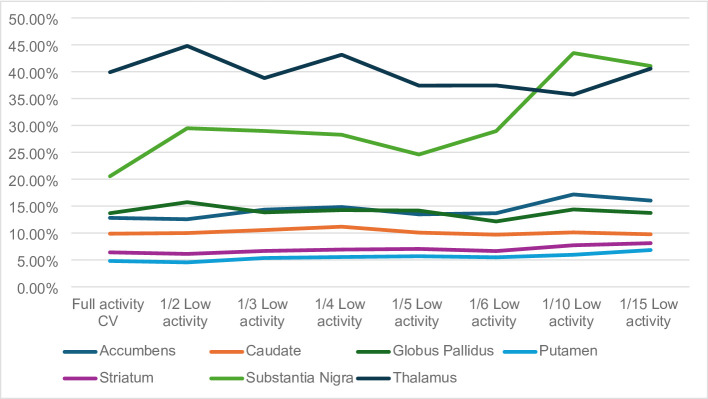
Summary display of the coefficients of variation (CVs) obtained for the low activity [11 C]-(+)-PHNO on BPND

The analysis of the coefficient of variation (CV) revealed that the substantia nigra and the thalamus presented the highest values for the Full activity datasets (20.55% and 39.90%, respectively). The CVs, for the Full activity datasets of all other structures were below 20%, as presented in Supplementary Table 1. A summary display of the CVs is present in Fig. 1.

When investigating the bias (as an absolute measure) present in the low activity simulations, it was noted that the 1/10 and the 1/15 low activity simulations consistently produced the highest bias for all structures. The 1/2 and 1/3 low activity simulations produced the lowest bias for the caudate, globus pallidus and thalamus. For the accumbens, putamen, striatum and substantia nigra, the 1/5 simulation produced the lowest bias, as indicated in Table 2.

**Table 2:** Bias calculated between the low activity simulated datasets and the full activity datasets, when investigating the impact of low activity [^11^C]-(+)PHNO on BP_ND_

	1/2 Low activity	1/3 Low activity	1/4 Low activity	1/5 Low activity	1/6 Low activity	1/10 Low activity	1/15 Low activity
Accumbens	-0.049	0.059	0.054	0.036	0.106	0.238	0.328
Caudate	-0.050	-0.005	0.012	0.046	0.051	0.132	0.170
Globus Pallidus	0.009	0.069	0.017	0.154	0.143	0.251	0.387
Putamen	-0.050	-0.020	-0.023	-0.006	0.018	0.058	0.119
Striatum	-0.053	-0.013	-0.011	0.012	0.035	0.103	0.155
Substantia Nigra	0.028	0.030	0.031	0.022	0.077	0.082	-0.057
Thalamus	-0.029	-0.016	-0.043	-0.019	-0.030	-0.030	-0.032

Out of all the structures investigated, the substantia nigra and the thalamus were the only ones that did not follow a normal distribution. Moreover, with the exception of the putamen and the striatum, no statistically significant differences were observed when comparing the 1/2, 1/3, 1/4, 1/5, 1/6 low activity simulations with the Full activity dataset.

**Fig. 2:**
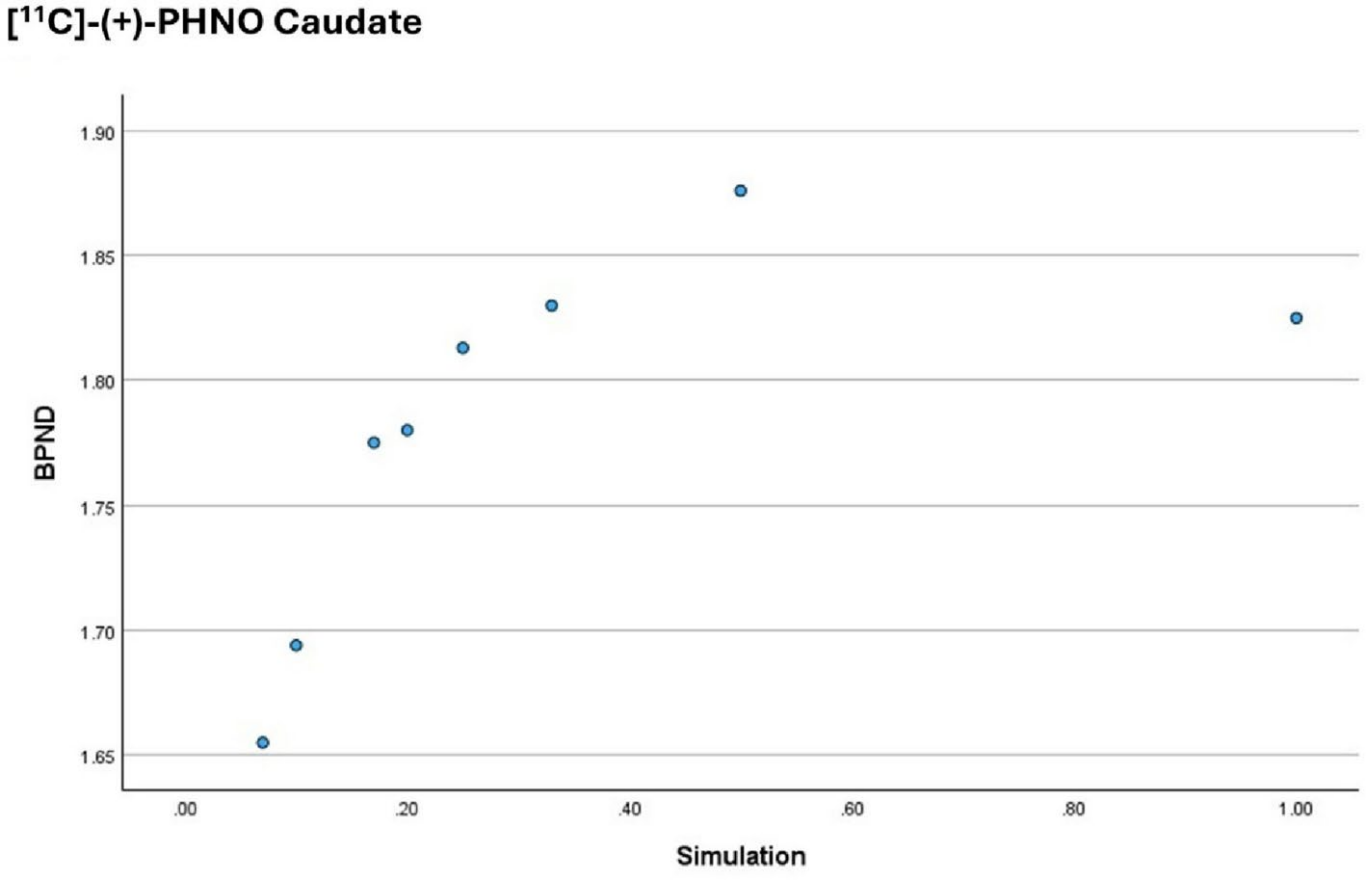
BP_ND_ obtained from the [^11^C]-(+)-PHNO in the Caudate, per activity simulation. The graph demonstrates that there is no correlation between the dependent variable and the independent variable

Figure 2 plots the outcome measure for the [^11^C]-(+)-PHNO on the y axis (the BP_ND_ parameter) against the activity simulations on the x axis (1 is the equivalent to the Full activity dataset and 0.2 is the equivalent to the 1/5 low activity simulation), for the Caudate.

The data indicates that above 50% of the injected activity, there is no correlation between the outcome measure (BP_ND_) and the simulation performed, for the [^11^C]-(+)-PHNO. However, below this threshold, the outcome measure appears to decrease with the injected activity. Moreover, the graph also shows a significant decrease in the value of the outcome measure for simulations mimicking significant low activity scenarios, such as the 1/10 and the 1/15 simulations.

### Findings of low activity [^11^C]UCB-J on BP_ND_

**Fig. 3:**
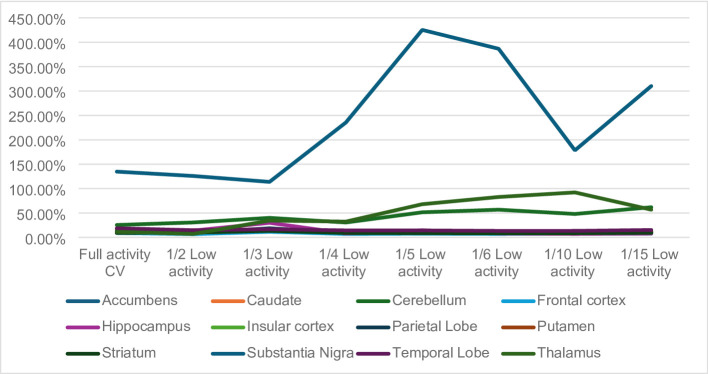
Summary display of the coefficients of variation (CVs) obtained for the low activity [11 C]UCB-J on BPND

During the analysis of the coefficient of variation (CV) it was noted that the cerebellum and the substantia nigra presented the highest values for the Full activity datasets (25.47% and 134.80%, respectively). The CVs, for the Full activity datasets of all other structures were below 20%, as presented in Supplementary Table 2. A summary display of the CVs is present in Fig. 3.

When investigating the bias present in the low activity simulations, it was noted that the 1/15 low activity simulations produced the highest bias for all structures, with the exception of the thalamus. For the thalamus, the 1/6 low activity simulations produced the highest bias of 0.939, closely followed by the 1/10 and 1/15 low activity simulations with biases of 0.937 and 0.541, respectively. The 1/2 and 1/3 low activity simulations produced the lowest bias for all structures (Table 3).

**Table 3:** Bias calculated between the low activity simulated datasets and the Full activity datasets, when investigating the impact of low activity [^11^C]UCB-J on BP_ND_

	1/2 Low activity	1/3 Low activity	1/4 Low activity	1/5 Low activity	1/6 Low activity	1/10 Low activity	1/15 Low activity
Accumbens	0.453	0.670	0.867	1.045	1.152	1.358	1.536
Caudate	0.154	-0.065	0.320	0.394	0.523	0.653	0.826
Cerebellum	0.166	0.468	0.536	0.735	0.815	0.851	1.037
Frontal cortex	0.232	0.290	0.450	0.547	0.604	0.720	0.878
Hippocampus	-0.046	0.162	0.532	0.686	0.822	0.888	1.008
Insular cortex	0.100	0.601	0.726	0.884	0.952	1.113	1.248
Parietal Lobe	0.287	0.271	0.568	0.677	0.758	0.869	1.006
Putamen	0.207	0.284	0.478	0.634	0.731	0.871	1.035
Striatum	0.208	0.234	0.453	0.588	0.690	0.834	1.002
Substantia Nigra	0.133	0.182	0.345	0.334	0.641	0.649	0.809
Temporal Lobe	0.130	0.540	0.664	0.804	0.867	1.052	1.193
Thalamus	0.126	0.351	0.419	0.817	0.939	0.937	0.541

**Fig. 4:**
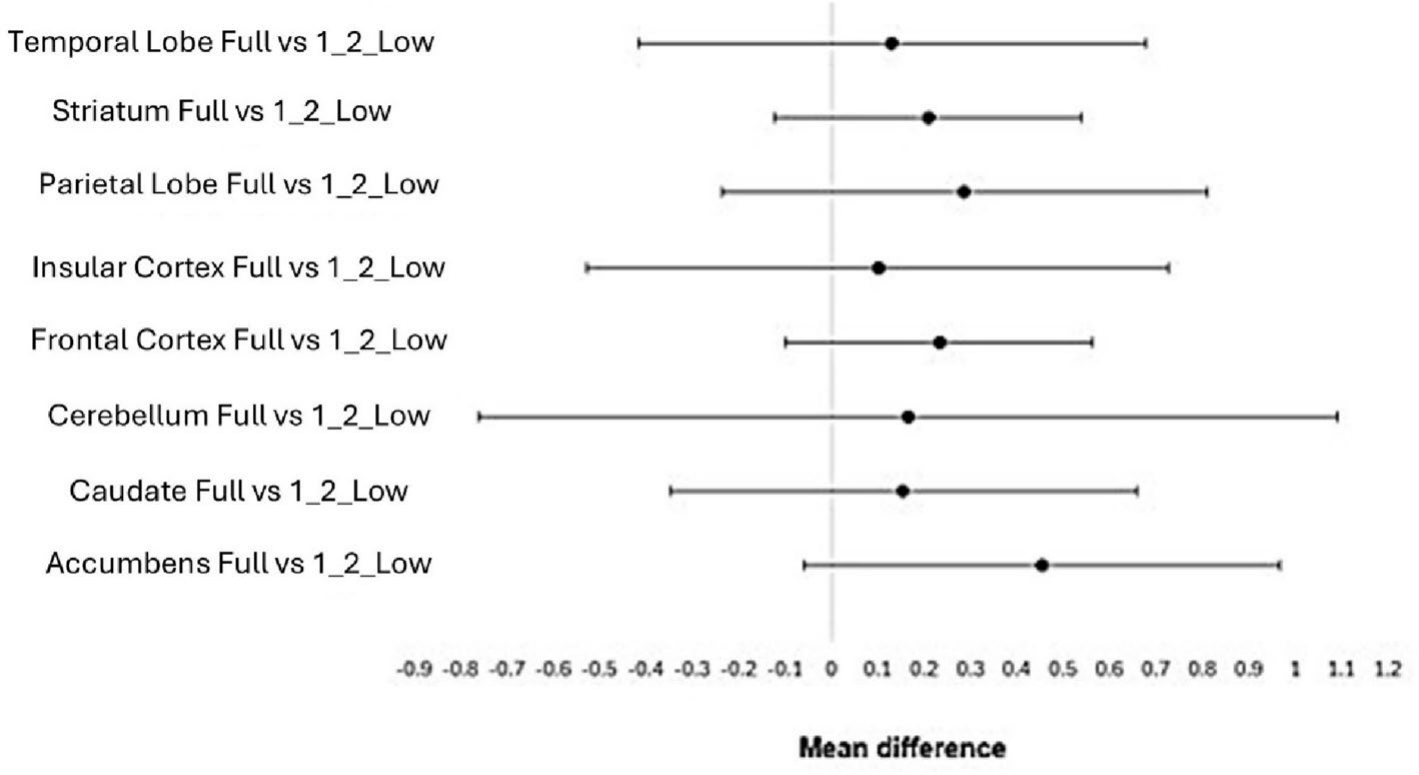
Visual representation of no statistically significant differences present when comparing the Full activity dataset with the 1/2 low activity dataset, for the parametric data

Out of all the structures investigated, the hippocampus, putamen, substantia nigra and thalamus were the ones that did not follow a normal distribution. No statistically significant differences were found when comparing the 1/2 low activity with the full activity datasets, for all the structures, as indicated in Fig. 4.

Moreover, with the exception of the accumbens, the remaining brain structures also did not reveal any statistically significant differences when the 1/3 low activity and the full activity datasets were compared.

**Fig. 5:**
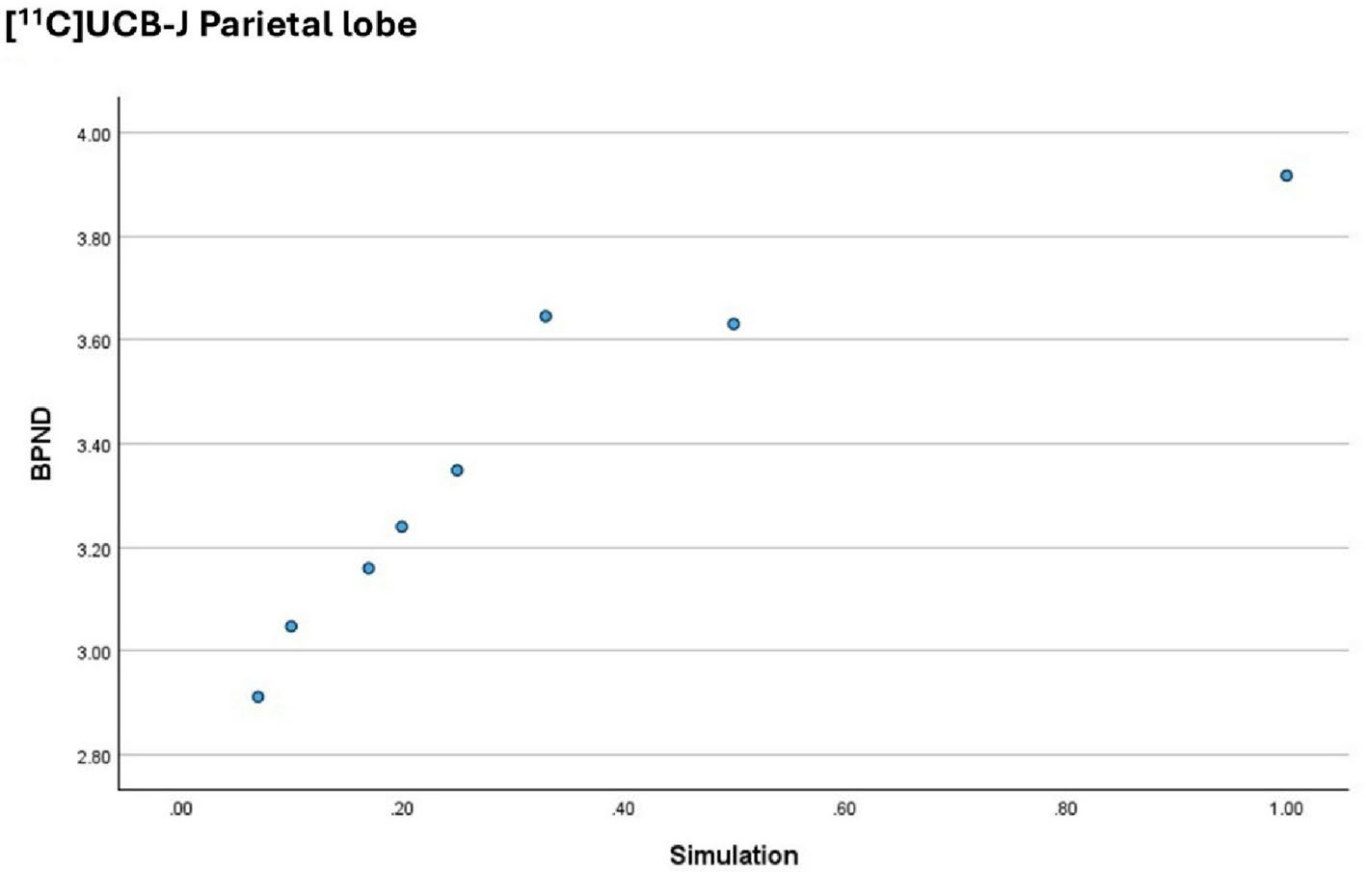
BP_ND_ obtained from the [^11^C]UCB-J in the Parietal lobe, per activity simulation. The graph demonstrates that there is no correlation between the dependent variable and the independent variable

Figure 5 plots the outcome measure for the [^11^C]UCB-J on the y axis (the BP_ND_ parameter) against the activity simulations on the x axis (1 is the equivalent to the Full activity dataset and 0.2 is the equivalent to the 1/5 low activity simulation), for the Parietal lobe.

The plot indicates that above 33% of the injected activity, there is no correlation between the outcome measure (BP_ND_) and the simulation performed, for the [^11^C]UCB-J. Below this threshold, the outcome measure appears to decrease with the injected activity, for this radiopharmaceutical.

### Findings of low activity [^11^C]UCB-J on SUV_R_

**Fig. 6:**
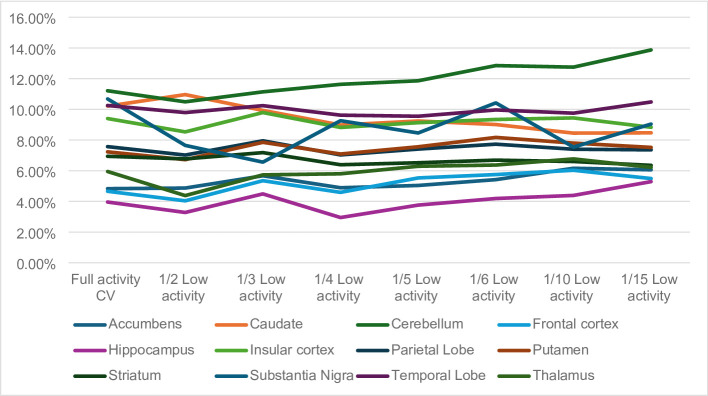
Summary display of the coefficients of variation (CVs) obtained for the low activity [11 C]UCB-J on SUVR

During the analysis of the coefficient of variation (CV) it was found that none of the structures presented variations above 15% (as indicated in Supplementary Table 3). A summary display of the CVs is present in Fig. 6. The structures with the highest CVs for the full activity datasets were the caudate (10.21%), cerebellum (11.21%), substantia nigra (10.68%) and temporal lobe (10.25%).

An analysis of the bias revealed that the 1/2 low activity simulation consistently produced the lowest bias for all structures (Table 4). With the exception of the substantia nigra, the highest bias for the remaining structures was found with the 1/15 low activity simulation. For the substantia nigra, the highest bias was found in the 1/10 low activity dataset (0.076), with the second highest bias being the one obtained in the 1/15 low activity dataset (0.074). Similarly to what was found when examining the bias obtained for the [^11^C]UCB-J BP_ND_, the less counts will be available in the brain structures, and the higher the bias will be.

**Table 4:** Bias calculated between the low activity simulated datasets and the full activity datasets, when investigating the impact of low activity [^11^C]UCB-J on SUV_R_

	1/2 Low activity	1/3 Low activity	1/4 Low activity	1/5 Low activity	1/6 Low activity	1/10 Low activity	1/15 Low activity
Accumbens	0.017	0.086	0.159	0.263	0.318	0.433	0.555
Caudate	0.031	0.106	0.157	0.262	0.324	0.437	0.598
Cerebellum	0.031	0.090	0.158	0.203	0.233	0.310	0.409
Frontal cortex	0.017	0.089	0.137	0.228	0.273	0.360	0.484
Hippocampus	0.006	0.079	0.106	0.209	0.260	0.296	0.387
Insular cortex	0.014	0.084	0.137	0.229	0.271	0.368	0.481
Parietal Lobe	0.018	0.082	0.133	0.227	0.279	0.352	0.472
Putamen	0.024	0.113	0.173	0.296	0.362	0.465	0.599
Striatum	0.025	0.106	0.165	0.279	0.341	0.450	0.591
Substantia Nigra	0.027	0.031	0.066	0.047	0.076	0.076	0.074
Temporal Lobe	0.011	0.075	0.133	0.211	0.249	0.346	0.463
Thalamus	0.026	0.081	0.105	0.167	0.203	0.225	0.269

**Fig. 7:**
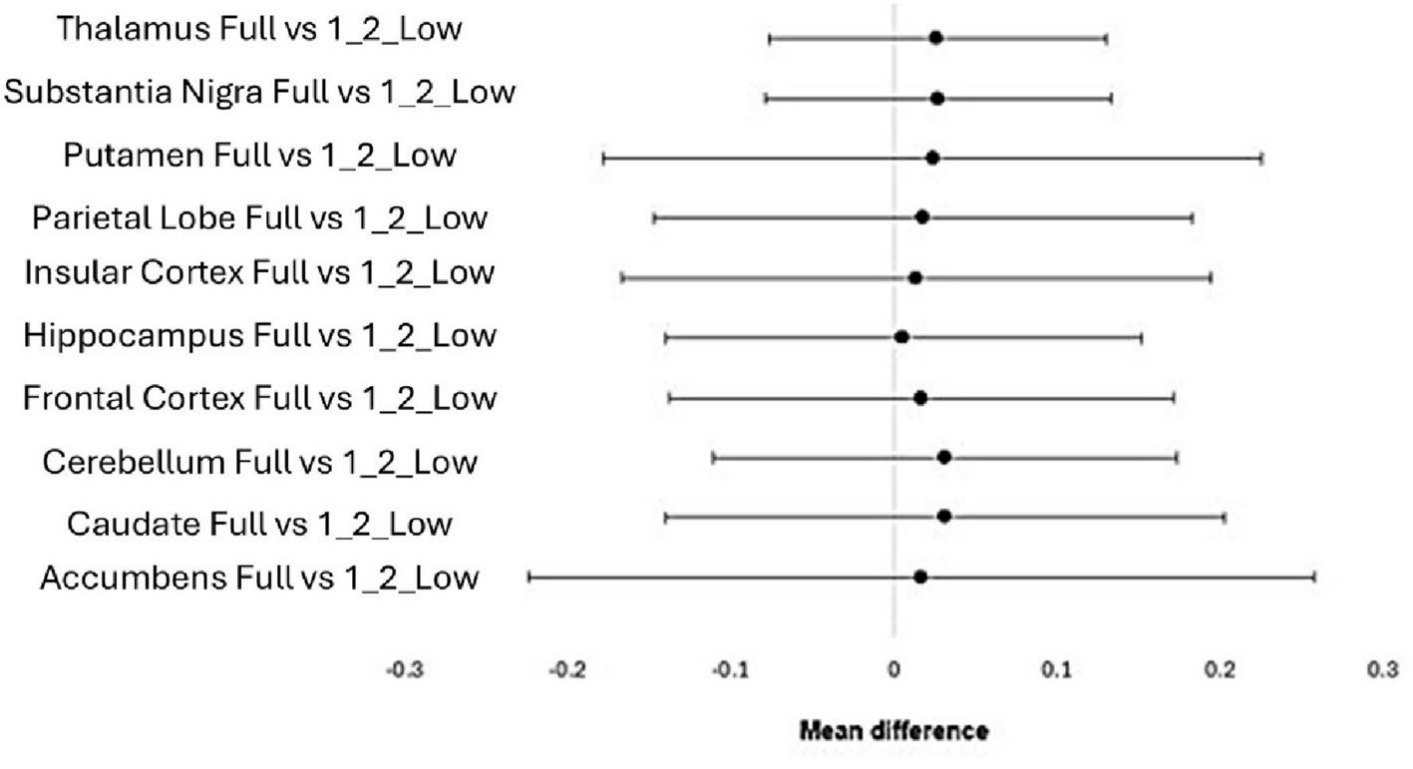
Visual representation of no statistically significant differences present when comparing the Full activity dataset with the 1/2 low activity dataset, for the parametric data

**Fig. 8:**
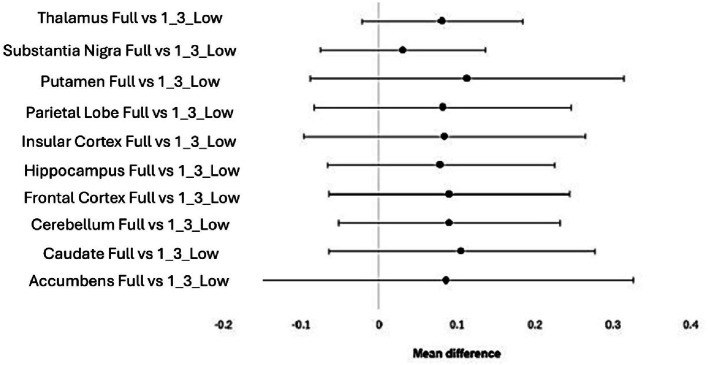
Visual representation of no statistically significant differences present when comparing the full activity dataset with the 1/3 low activity dataset, for the parametric data

Out of all the structures investigated, the striatum and the temporal lobe were the only ones that did not follow a normal distribution. No statistically significant differences were found when comparing the 1/2 and 1/3 low activity datasets with the full activity datasets, for all the structures, as indicated in Figs. 7 and 8 respectively.

Moreover, with the exception of the cerebellum and the thalamus, the remaining brain structures also did not reveal any statistically significant differences when the 1/4 low activity and the full activity datasets were compared.

**Fig. 9:**
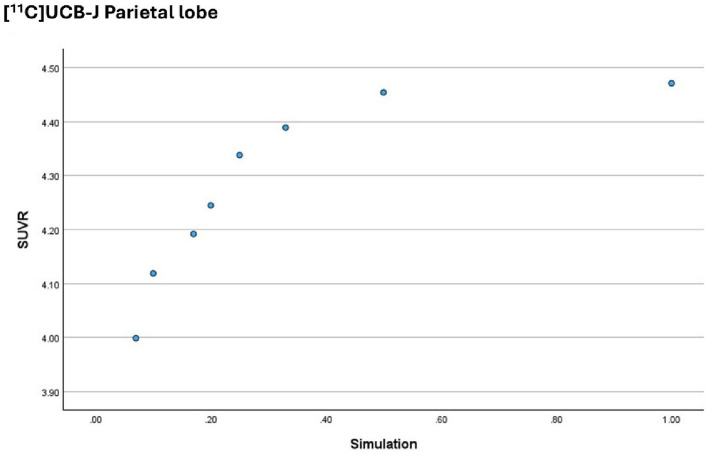
SUV_R_ obtained from the [^11^C]UCB-J in the Parietal lobe, per activity simulation. The graph demonstrates that there is no correlation between the dependent variable and the independent variable

Figure 9 plots the outcome measure for the [^11^C]UCB-J on the y axis (the SUV_R_ parameter) against the activity simulations on the x axis (1 is the equivalent to the Full activity dataset and 0.2 is the equivalent to the 1/5 low activity simulation), for the Parietal lobe.

The plot indicates that above 33% of the injected activity, there is no correlation between the outcome measure (SUV_R_) and the simulation performed, for the [^11^C]UCB-J. Below this threshold, the outcome measure appears to decrease with the injected activity, for this radiopharmaceutical.

## **Discussion**

### Impact of low activity [^11^C]-(+)-PHNO on BP_ND_

Small structures, such as the substantia nigra, are more susceptible to low activity simulations, particularly those in which the number of counts is significantly reduced. Whilst the transaxial spatial resolution of the GE SIGNA PET-MR scanner is of the order of 4 mm near the centre of the field of view, the substantia nigra is a small structure with a thickness of 5.1 ± 0.89 mm noted in health volunteers [17, 18]. Therefore, the visibility of such a small structure will be substantially impacted by the amount of radiopharmaceutical administered and the movement during scan acquisition.

**Fig. 10:**
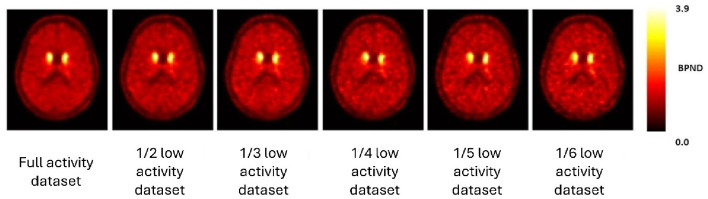
BP_ND_ axial images representative of the full dataset and low dose datasets, 1/2 to 1/6

In the case of the thalamus, when simulating low activity scenarios of 1/2, 1/3, 1/4, 1/5, 1/6, 1/10 and 1/15, the CV ranged between a minimum of 35.75% and a maximum of 44.79%. In comparison to the CV obtained for the Full activity dataset, this variation only equated to a difference of a maximum of approximately 5%, which can be accepted when conducting these simulations. This, however, demonstrates that the high CV is not related to the simulation method itself but rather with the brain structure under investigation and the radiopharmaceutical uptake. Figure 10 demonstrates the uptake of [^11^C]-(+)-PHNO in the brain, across the low activity datasets.

**Fig. 11:**
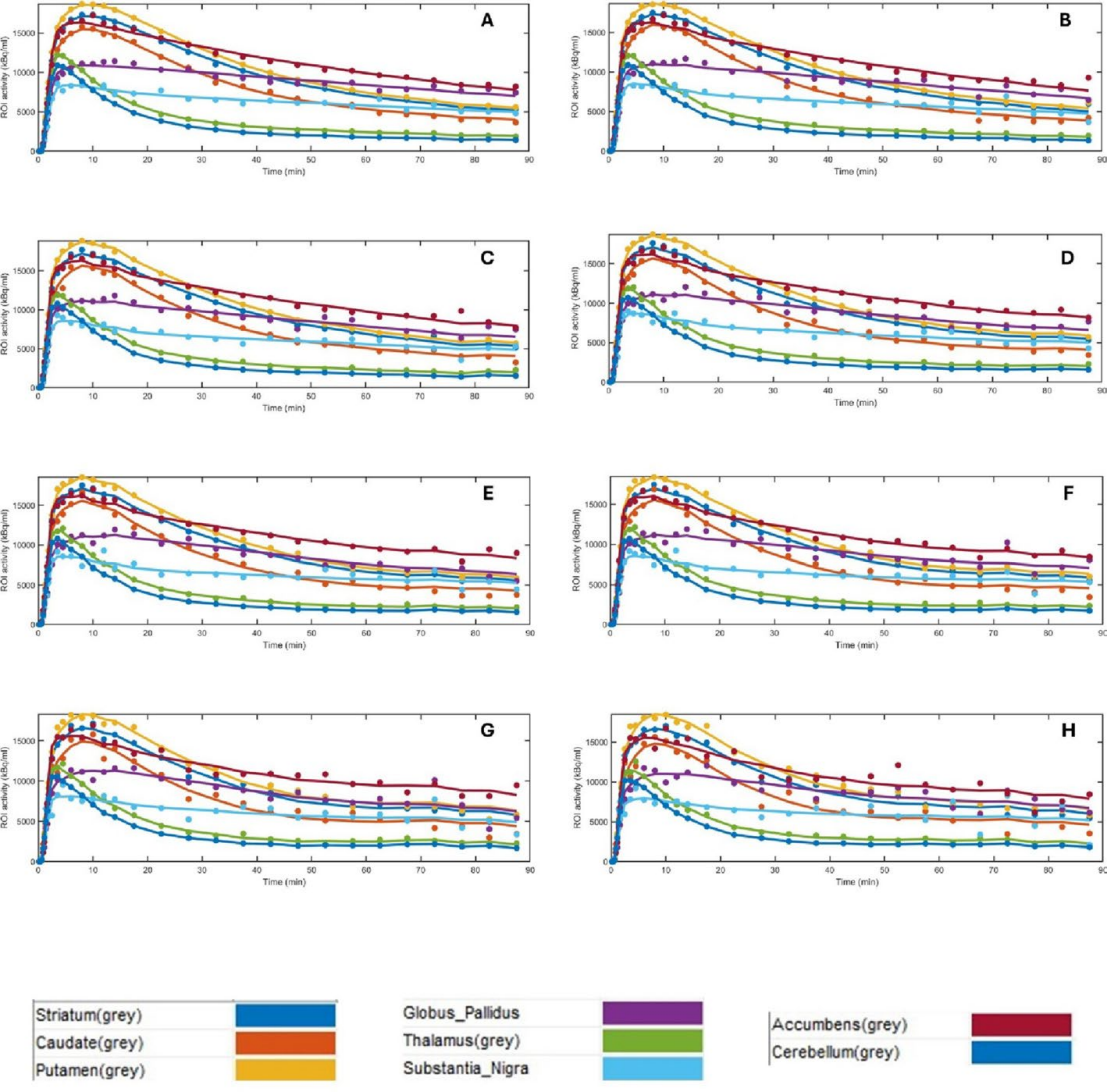
Time activity curves (TACs) obtained for the striatum, caudate, putamen, globus pallidus, thalamus, substantia nigra and accumbens, for one participant dataset. **A**- TACs from those datasets representative of Full activity. **B**- TACs those datasets representative of 1/2 of the administered activity. **C**- TACs those datasets representative of 1/3 of the administered activity; **D**- TACs those datasets representative of 1/4 of the administered activity; **E**- TACs those datasets representative of 1/5 of the administered activity; **F**- TACs those datasets representative of 1/6 of the administered activity; **G**- TACs those datasets representative of 1/10 of the administered activity; **H**- TACs those datasets representative of 1/15 of the administered activity

In the case of the putamen, the 1/5 simulation produced the smallest bias, followed by the 1/6, 1/3, 1/4 and 1/2 simulations. A similar pattern was noted in the striatum, with the 1/4 simulation producing the smallest bias, followed by the 1/5, 1/3, 1/6 and 1/2 simulations. Although the putamen and the striatum presented statistically significant differences when comparing the 1/2, 1/10 and 1/15 with the Full activity datasets, a visual inspection of the graphics in Fig. 8 reveals that there is minimal to no change in the shape of the time activity curves for the 1/2 low activity and the Full activity datasets (Fig. 11).

### Impact of low activity [^11^C]UCB-J on BP_ND_

In the case of the cerebellum, when simulating low activity scenarios of 1/2, 1/3 and 1/4, the CV ranged between a minimum of 30.60% up to a maximum of 40.07%. In comparison to the CV obtained for the Full activity dataset, this variation equated to a difference of a maximum of 14.6%. When simulating low activity scenarios of 1/5, 1/6, 1/10 and 1/15, the CV ranged between a minimum of 48.06% and a maximum of 61.82%, in comparison with the full activity dataset. This variation equated to a difference of a maximum of 36.35%. This demonstrates that the wider variability is related to the brain structure itself and the uptake of the radiopharmaceutical in the region, and that this effect is exacerbated under very low activity simulations.

In the case of the substantia nigra, the CV results obtained appear to be skewed, affecting not only the low activity simulation datasets but also in the full activity dataset. The minimum BP_ND_ values obtained for all the datasets (low activity and full activity) are negative. This is further substantiated by the fact that the CV obtained for the full activity dataset was of the order of 134.8%, and the CVs obtained for the 1/2 to 1/15 simulations range between a minimum of 113.8% to maximum of 425.2%.

These findings prompted a more in-depth review of the BP_ND_ data obtained for each participant, at each low activity simulation. Whilst a negative outcome measure can potentially be present for low activity datasets due to the lack of sufficient counts in the brain regions, the BP_ND_ should never be negative for the full activity datasets. The data review revealed that, for two out of the five participants, the BP_ND_ obtained for the full activity dataset was negative. If the data from these participants had been removed, then the overall CV of the Full activity dataset would drop to 17.11% and the CV for the 1/2 activity simulation would drop to 49.74%. Additionally, for the Full activity and the 1/2 activity datasets, the minimum BP_ND_ value would also remain positive. The CV for the 1/3 to 1/15 low activity simulations would, however, remain high.

**Fig. 12:**
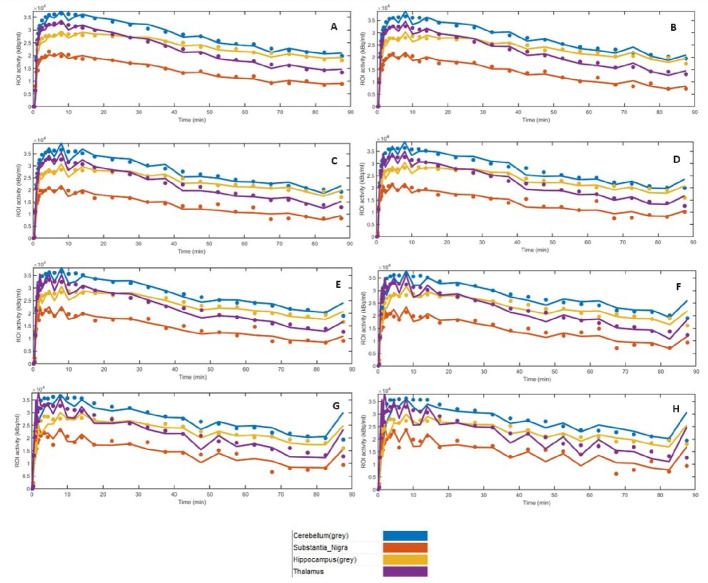
Time activity curves (TACs) obtained for the cerebellum, substantia nigra, hippocampus and thalamus, for one participant. **A**- TACs from those datasets representative of Full activity. **B**- TACs those datasets representative of 1/2 of the administered activity. **C**- TACs those datasets representative of 1/3 of the administered activity; **D**- TACs those datasets representative of 1/4 of the administered activity; **E**- TACs those datasets representative of 1/5 of the administered activity; **F**- TACs those datasets representative of 1/6. **G**- TACs those datasets representative of 1/10; **H**- TACs those datasets representative of 1/15 dataset

Figure 12 presents the time activity curves, for all the low activity simulations and the full activity dataset, for the cerebellum, substantia nigra, hippocampus and thalamus.

In the case of the hippocampus, the CV obtained for the 1/2 low activity dataset is very similar to that of the full activity dataset (13.92% and 17.18%, respectively). However, for the 1/6, 1/10 and 1/15 low activity datasets, there is a substantial decrease of the CV (8.07%, 7.22% and 8.08%, respectively). This drop in CV may be due to difficulty in identifying counts in the hippocampus, when the activity is significantly reduced. The thalamus, however, displays the opposite case to that observed for the hippocampus. Whilst the 1/2 low activity and Full activity datasets present very similar CVs (7.08% and 11.42%, respectively), there is a substantial increase in CVs when the 1/3, 1/4, 1/5, 1/6, 1/10 and 1/15 simulations are performed (34.52%, 32.41%, 68.06%, 82.85%, 92.21% and 56.80%, respectively).

### Impact of low activity [^11^C]UCB-J on SUV_R_

The highest CVs detected were in line with those observed for the [^11^C]UCB-J BP_ND_ results, as both the cerebellum and substantia nigra had been noted as having the highest CVs, for the full activity datasets. This further sustains the premise that the wider variability is partially related to the brain structures themselves and the radiopharmaceutical uptake in that region. The low CVs obtained for the full activity datasets demonstrate that there is less variability around the mean of the SUV_R_, suggesting the stability and reliability of this outcome measure.

**Fig. 13:**
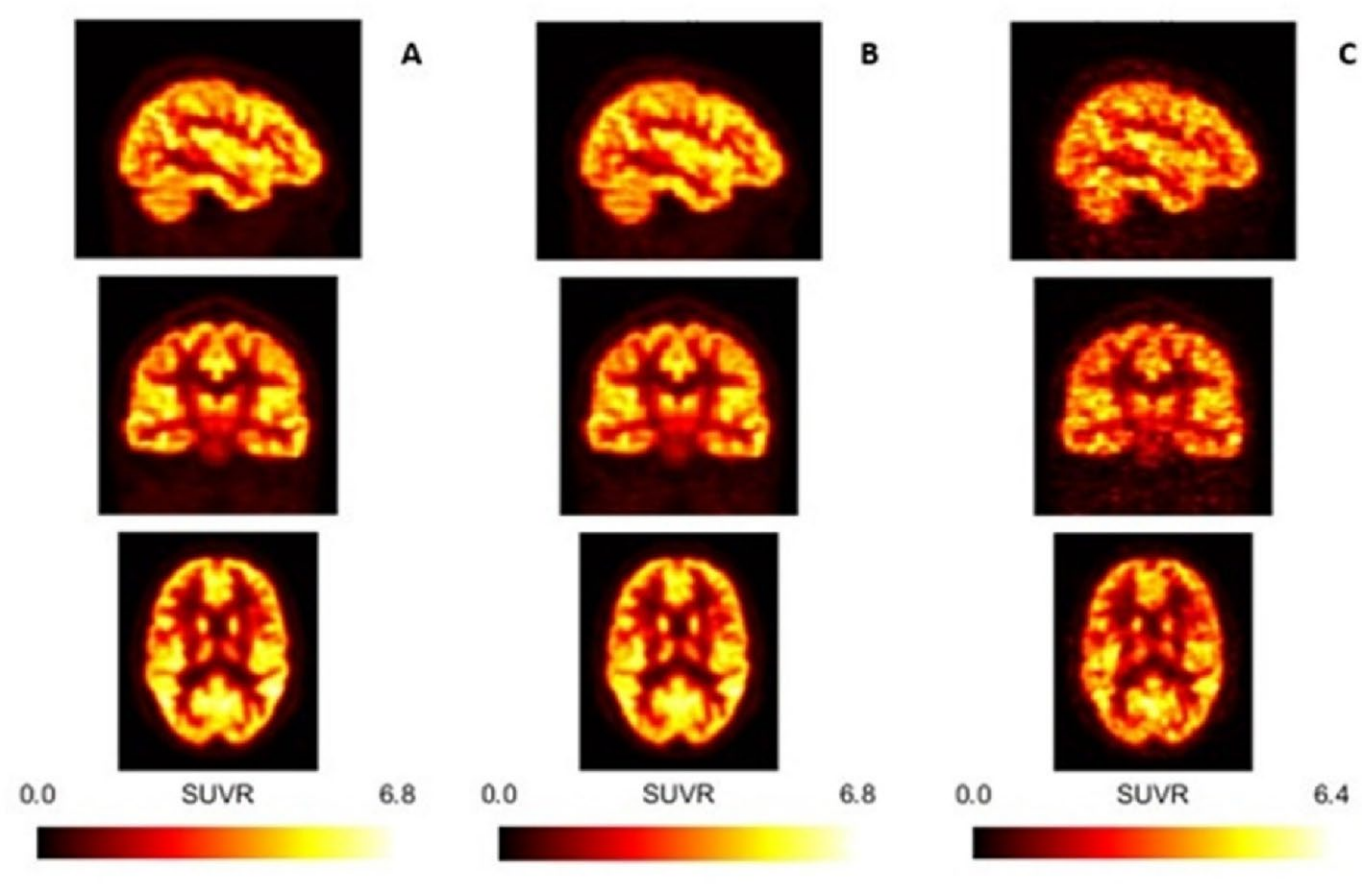
SUV_*R*_ images obtained from three different datasets from the same participant (the top images are sagittal views; the middle images; are coronal views and the lower images are axial views). Dataset A is representative of the Full activity dataset; dataset B is representative of the 1/2 low activity dataset; dataset C is representative of the 1/15 low activity dataset.

Figure 13 presents the SUV_R_ parametric images for the full activity, 1/2 and 1/15 low activity datasets

Moreover, when analysing the range of the CVs obtained for all the low activity simulations, none of the structures presented variations above 4%. The structures with the highest variation of CVs for the low activity simulations were the substantia nigra (3.86%), cerebellum (3.38%), caudate (2.51%) and thalamus (2.39%). This finding is also in line with that observed for the [^11^C]UCB-J BP_ND_ results, as the cerebellum and substantia nigra had been noted as having the highest variation of CVs, for the low activity datasets. Overall, the low CVs obtained when the various regimens of the low activity datasets are compared, further sustains the premise that the SUV_R_ is a robust outcome measure when investigating low activity scenarios.

### Overall discussion

When comparing the low activity simulations and BP_ND_ analysis conducted for the [^11^C]-(+)-PHNO datasets with that which was conducted for the [^11^C]UCB-J, it can be understood that the methodology worked best in the latter dataset. In the [^11^C]UCB-J BP_ND_ analysis there was a consistent tendency for the 1/2 and/or 1/3 low activity simulations to produce the lowest bias. With the [^11^C]-(+)-PHNO BP_ND_ analysis however, this pattern was not predominant. Whilst some structures did show the above pattern, the putamen and striatum were clear outliers. Similarly, whilst the [^11^C]UCB-J BP_ND_ analysis did not reveal any statistically significant differences when the 1/2 low activity simulation was compared to the full activity dataset, there were structures within the [^11^C]-(+)-PHNO BP_ND_ analysis that revealed statistically significant differences for the same comparison.

Additionally, when comparing the low activity simulations and outcome measures for the [^11^C]UCB-J BP_ND_ and [^11^C]UCB-J SUV_R_ analysis, it can be perceived that the methodology worked best in the later. Whilst in the BP_ND_ analysis there was a tendency for the 1/2 and/or 1/3 low activity simulations to produce the lowest bias, in the SUV_R_ the 1/2 low activity simulation consistently produced the lowest bias for all structures. Similarly, whilst the BP_ND_ analysis did not reveal any statistically significant differences when the 1/2 low activity simulation was compared to the Full activity dataset, the SUV_R_ analysis did not reveal any statistically significant differences when the Full activity dataset was compared to the 1/2 and 1/3 low activity simulations. It is therefore hypothesised that the simulation and analysis methodologies used in this study are best suited when investigating SUV_R_, rather than BP_ND_.

## **Conclusion**

Whilst the data suggests that the efficacy of the investigation is highly dependent on the algorithm used to reconstruct the images, the outcome measure and the radiopharmaceutical, it can be assumed that the methodology used is well suited for investigating low activity scans for tracers with cortical and striatal uptake, when the outcome measure assessed is the BP_ND_ or the SUV_R_.

Importantly, for the [^11^C]UCB-J radiopharmaceutical, the data shows data the injectable activity can be decreased to 1/3 of the original administered activity, without a compromise to the clinical outcome measure SUV_R_.

Activity optimisation and low activity imaging are aligned with the principles of justification and optimisation from the ALARA and “As Low as Reasonably Practicable” (ALARP) guidance, as well as the recommendations of the International Commission on Radiological Protection (ICRP) [19, 20]. Moreover, activity optimisation and low activity imaging are intrinsically related with sustainability and, as Currie et al. (2024) indicated, the optimisation of acquisition protocols is a potential approach for fostering environmental sustainability [21]. In the case of radiopharmaceuticals specifically produced for research purposes, ensuring their sustainable production and consumption, in central and local radiopharmacies, is directly related with the United Nations’ Sustainable Development Goal (SDG) 12 – Responsible consumption and production. Taking action to combat climate change and its impacts, through optimising and justifying Nuclear Medicine procedures is in line with SDG 13 – Climate change [22].

Additionally, low activity imaging is also related to social sustainability. Ensuring the healthy lives and promoting well-being of patients (particularly those living with Neurodevelopmental disorders), carers and family members who attend nuclear medicine examinations, through a reduction in radiopharmaceutical administration and radiation exposure is directly correlated with the SDG 3 – Good health and well-being. Similarly, promoting the well-being of professionals who work within the field of Nuclear Medicine, through a decrease of occupational exposure levels is directly correlated with this SDG.

The authors acknowledge two limitations with the present experiment. The first is related to the simulation technique used whereby frames were shortened by inserting delays and, therefore, the activity concentration may not represent the true count average over the original frame duration. This strategy has the potential to change the mid-times of the time-activity curves and therefore bias kinetic modelling. However, in order to limit the impact of this limitation, all delays were consistently inserted at the start of the frames thereby ensuring that, if a bias was present, it would be consistent within all the datasets and minimally affect the quantitative results. The second limitations which was noted was that this experiment was only conducted in datasets reconstructed with OSEM. While no other algorithms were explored in this study, they will be in future work.

## Supplementary Information

Below is the link to the electronic supplementary material.

Supplementary Material 1.

## Data Availability

The datasets generated and/or analysed during the current study are not publicly available are available from the corresponding author on reasonable request.

## Abbreviations

ADHD: Attention deficit hyperactivity disorder
ALARA: As low as reasonably achievable
ALARP: As low as reasonably practicable
ASD: Autism spectrum disorder
BPD: Bipolar disorder
BP_ND_: Binding potential relative to non-displaceable volume
CV: Coefficient of variation
DFOV: Display field of view
DSM: 5_-_Diagnostic and Statistical Manual of Mental Disorders fifth edition
FSPGR: Fast spoiled gradient-echo sequence
GCP: Good clinical practice
ICRP: International Commission on Radiological Protection
ID: Intellectual disability
MR: Magnetic resonance
NDDs: Neurodevelopmental disorders
NHS: National Health Service
PET: Positron emission tomography
SDG: Sustainable development goal
SRTM: Single reference tissue model
SUV_R_: Standard uptake volume ratio
TACs: Time activity curves
TOF: Time-of-flight
