# Supplementary material for: Evaluation of the impact of low activity imaging in [11C]-(+)-PHNO and [11C]UCB-J PET-MR scans
Source: EJNMMI Phys. 2026 Mar 10;13:36. doi: 10.1186/s40658-026-00850-y (PMC13086985; doi:10.1186/s40658-026-00850-y)
Supplement: Supplementary file 1 — Supplementary Material 1. [file 40658_2026_850_MOESM1_ESM.docx]

Supplementary tables

Supplementary Table 1 Coefficients of variation (CVs) obtained when investigating the impact of low activity [^11^C]-(+)-PHNO on BP_ND_

|  | **Full activity CV** | **1/2 Low activity** | **1/3 Low activity** | **1/4 Low activity** | **1/5 Low activity** | **1/6 Low activity** | **1/10 Low activity** | **1/15 Low activity** |
| --- | --- | --- | --- | --- | --- | --- | --- | --- |
| **Accumbens** | 12.81% | 12.56 % | 14.34% | 14.83% | 13.47% | 13.68% | 17.17% | 16.03% |
| **Caudate** | 9.87% | 9.99% | 10.55% | 11.17% | 10.07% | 9.69% | 10.11% | 9.76% |
| **Globus Pallidus** | 13.68% | 15.73 % | 13.84% | 14.26% | 14.17% | 12.15% | 14.38% | 13.73% |
| **Putamen** | 4.80% | 4.54% | 5.35% | 5.53% | 5.68% | 5.48% | 5.95% | 6.83% |
| **Striatum** | 6.40% | 6.11% | 6.66% | 6.92% | 7.04% | 6.64% | 7.72% | 8.11% |
| **Substantia Nigra** | 20.55% | 29.48 % | 28.97% | 28.27% | 24.60% | 28.95% | 43.47% | 41.05% |
| **Thalamus** | 39.90% | 44.79 % | 38.81% | 43.15% | 37.41% | 37.44% | 35.75% | 40.57% |

Supplementary Table 2 Coefficients of variation (CVs) obtained when investigating the impact of low activity [^11^C]UCB-J on BP_ND_

|  | **Full activity CV** | **1/2 Low activity** | **1/3 Low activity** | **1/4 Low activity** | **1/5 Low activity** | **1/6 Low activity** | **1/10 Low activity** | **1/15 Low activity** |
| --- | --- | --- | --- | --- | --- | --- | --- | --- |
| **Accumbens** | 13.10% | 10.11% | 11.89% | 9.21% | 8.15% | 7.62% | 7.92% | 7.82% |
| **Caudate** | 12.26% | 14.50% | 16.33% | 11.31% | 9.96% | 12.63% | 12.29% | 12.13% |
| **Cerebellum** | 25.47% | 30.65% | 40.07 % | 30.60 % | 51.48% | 56.81% | 48.06% | 61.82% |
| **Frontal cortex** | 9.09% | 6.69% | 11.53% | 7.09% | 7.69% | 7.21% | 7.80% | 9.58% |
| **Hippocampus** | 17.18% | 13.92% | 29.93% | 8.11% | 14.56% | 8.07% | 7.22% | 8.08% |
| **Insular cortex** | 16.74% | 13.58% | 14.56% | 12.27% | 12.50% | 11.60% | 12.10% | 11.83% |
| **Parietal Lobe** | 12.65% | 9.14% | 18.70% | 9.64% | 10.23% | 9.75% | 9.60% | 10.82% |
| **Putamen** | 9.62% | 9.69% | 14.15% | 10.25% | 10.40% | 10.19% | 9.90% | 11.32% |
| **Striatum** | 9.14% | 9.87% | 13.38% | 9.08% | 8.67% | 8.35% | 8.53% | 9.50% |
| **Substantia Nigra** | 134.8% | 125.9 % | 113.8% | 235.5% | 425.2% | 386.6% | 178.4% | 310.2% |
| **Temporal Lobe** | 18.72% | 14.76 % | 16.80% | 14.37% | 14.29% | 13.37% | 13.42% | 15.17% |
| **Thalamus** | 11.42% | 7.08% | 34.52% | 32.41% | 68.06% | 82.85% | 92.21% | 56.80% |

Supplementary Table 3 Coefficients of variation (CVs) obtained when investigating the impact of low activity [^11^C]UCB-J on SUV_R_

|  | **Full activity CV** | **1/2 Low activity** | **1/3 Low activity** | **1/4 Low activity** | **1/5 Low activity** | **1/6 Low activity** | **1/10 Low activity** | **1/15 Low activity** |
| --- | --- | --- | --- | --- | --- | --- | --- | --- |
| **Accumbens** | 4.83% | 4.87% | 5.67% | 4.89% | 5.04% | 5.42% | 6.16% | 6.06% |
| **Caudate** | 10.21% | 10.96% | 9.93% | 8.98% | 9.24% | 9.01% | 8.45% | 8.47% |
| **Cerebellum** | 11.21% | 10.49% | 11.14% | 11.63 % | 11.86% | 12.85% | 12.75% | 13.87% |
| **Frontal cortex** | 4.66% | 4.04% | 5.35% | 4.59% | 5.53% | 5.75% | 6.03% | 5.49% |
| **Hippocampus** | 3.96% | 3.28% | 4.49% | 2.95% | 3.76% | 4.19% | 4.39% | 5.29% |
| **Insular cortex** | 9.40% | 8.53% | 9.79% | 8.82% | 9.13% | 9.34% | 9.44% | 8.83% |
| **Parietal Lobe** | 7.57% | 7.02% | 7.95% | 7.03% | 7.41% | 7.73% | 7.40% | 7.36% |
| **Putamen** | 7.23% | 6.72% | 7.85% | 7.09% | 7.56% | 8.17% | 7.80% | 7.52% |
| **Striatum** | 6.94% | 6.77% | 7.18% | 6.39% | 6.52% | 6.69% | 6.60% | 6.35% |
| **Substantia Nigra** | 10.68% | 7.65% | 6.56% | 9.26% | 8.46% | 10.42% | 7.54% | 9.04% |
| **Temporal Lobe** | 10.25% | 9.79% | 10.24% | 9.62% | 9.55% | 9.96% | 9.75% | 10.48% |
| **Thalamus** | 5.95% | 4.38% | 5.73% | 5.80% | 6.29% | 6.37% | 6.77% | 6.22% |
